# Supplementary figures and images for: The influence of collection method on paleoecological datasets: In-place versus surface-collected fossil samples in the Pennsylvanian Finis Shale, Texas, USA
Source: PLoS One. 2020 Feb 11;15(2):e0228944. doi: 10.1371/journal.pone.0228944 (PMC7012410; doi:10.1371/journal.pone.0228944)

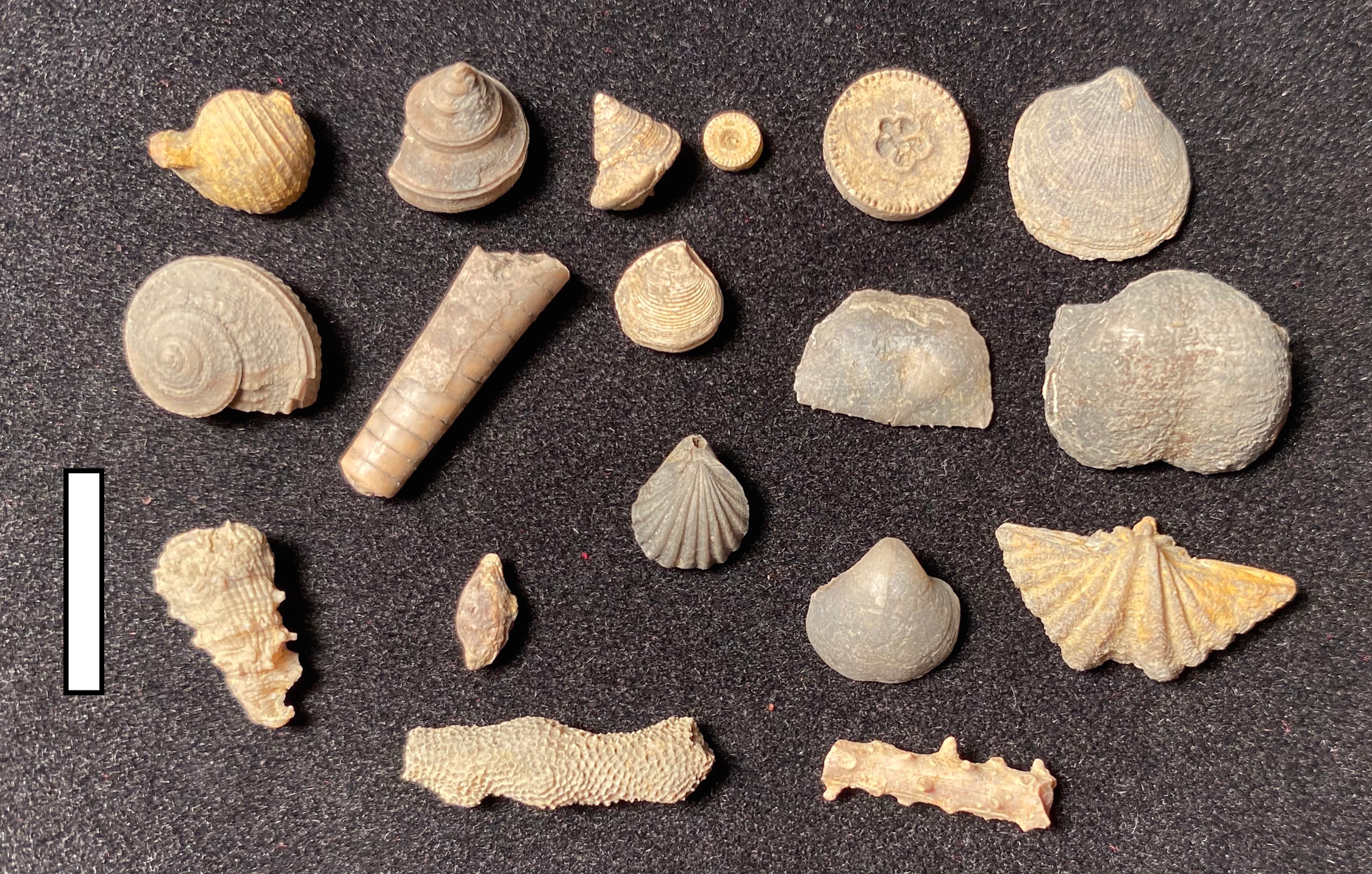

Supplement: S1 Fig — Scale bar is 1 cm. Top row, from left: Euphemites, Glabrocingulum sp., Phymatopleura, two crinoid columnals, Rhipidomella. Second row, from left: Glabrocingulum grayvillensis, Mooreceras, Astartella concentrica, Neochonetes, Marginifera. Third row, from left: Lophophyllidium, fusulinid, Hustedia, Crurithyris, Punctospirifer. Bottom row, from left: bryozoan colony fragment, echinoid spine fragment. (TIF) [file pone.0228944.s003.tif]
