# Supplementary material for: The influence of collection method on paleoecological datasets: In-place versus surface-collected fossil samples in the Pennsylvanian Finis Shale, Texas, USA
Source: PLoS One. 2020 Feb 11;15(2):e0228944. doi: 10.1371/journal.pone.0228944 (PMC7012410; doi:10.1371/journal.pone.0228944)
Supplement: Attachment — Submitted filename: Forcino and Stafford PLOS ONE Reviews Comment responses.docx [file pone.0228944.s006.docx]

PLOS ONE Reviews Comment responses:

***Review #1:***

Some doubts arise with regard to the fact that the smallest specimens were excluded from the studied samples (lines 209-219), especially that in lines 509-513 Authors show how important they are for interpretation (“A preferential loss of small, thin-shelled taxa in surface samples would produce misleading paleocommunity results”). In standard procedures we should focus on the whole material available (including the smallest taxa) to get the complete record of palaeoenvironmental conditions. “Smaller specimens would greatly increase sample size”, but do we know how the lack of them affect the comparisons presented in the paper? Are they still valid? This should be clarified in the text.

The reviews suggest that we need to examine fossils smaller than our cut off of 2mm. There are two reasons that we believe using fossils greater than 2mm is the best for this particular research project. (1) The goal of this paper is specifically to compare in-place fossil material to surface-collected material. Therefore, we wanted our bulk, in-place samples to be as comparable to the surface-collected samples as possible. The surface-collected sampling mimicked what would occur if a study were using this protocol. Picking up tiny specimens (< 2mm) off the muddy, weathered surface of an outcrop is unlikely (indeed, such specimens rarely occurred in the surface samples). The 2mm threshold for the in-place samples eliminated the potential size bias and allowed us to focus on other differences between the collection methods. (2) Examining paleocommunity differences between size fractions is part of the ongoing research in our lab. Sorting, identifying, and counting these tiny fossils is extraordinarily time consuming, and therefore would have added months to this specific project. Indeed, the smallest size fractions are underutilized in community paleoecology, and we believe this topic deserves its own attention as a research project, which is why we have active projects focusing on these very fossils.

The second general problem is a lack of comparisons with other sites, deposits, environments etc. I see that this is a plan for future works, but I think that it should be clarified earlier than in conclusions (in methods or in the beginning of the discussion section). Maybe there are some literature data to compare with, that could be included in the paper?!

This paper presents a specific case study that we hope spurs other researchers to choose their collection methods thoughtfully. We are not attempting to make generalizations about the results one might find in other localities, but we hope that other researchers could join in with replication of the study methods. Additionally, we are not drawing conclusions about the paleocommunity itself, in which case temporal and spatial context would be relevant.

The major contribution of this study is using complete communities with multivariate analyses to compare the paleocommunity results between the two different methods. There are no other studies that have done this to which we can compare our current results.

Geological background - I suggest to add figure with lithological logs in studied outcrops with indication of the sampling points

We added, “”See Forcino et al. [7] and Forcino et al. [35] for more detail about the Finis Shale outcrops at which we samples.” to the text for folks that want to know more about the detailed changes in the Finis Shale. Because it is lithologically homogeneous and because the sedimentology changes through the section do not influence the differences in the present study, we did not want to add extraneous text or figures.

L 49 – add “by” after influenced

Done

L 111, 112, 116, 125, 129, 134 – should be Fig 1A and Fig 1B instead of 1a and 1b

Done

L 244 – should be “multidimensional” instead of “multideminsional”

Done

L 289 – remove double “who” in the caption of Table 1.

Done

Clarify NA abbreviation.

The “NA”s were removed and replaced with greyed out boxes. The sentence “Subsampling was only necessary for surface samples, so there are no results listed for in-place samples.” was added to the table’s caption.

L 303-304 – Table 2: paired T-test, whereas t-test in the text; be consistent

Changed to “paired t-test” throughout.

L 324 – delete at in “letters at after”

Done

L 406 – delete “is”

Done

L 430 – remove dates from the citations

Done

L 532 – start with a new paragraph

Done

Fig. 1 – Check the scale and length of the histograms. They donʼt match the values cited in text (lines 113-116: slab/section in paleoecology articles 11/22 – on the figure > 25, other 18/32 – almost 40 etc.).

The figure and the text in the manuscript are both correct. The following text was added to the caption for figure 1, “The bar height in figure 1 represents the total number of methods used, not the total number of papers, because single papers sometimes used multiples methods.”

Fig. 2 – Show larger geographical context for international audience. Show the location on the map of the USA, or at least on its larger, more characteristic part. Put some coordinates or grid and more geographical/administrative names.

We added a map of the USA to replace the map of Texas to give context to where the outcrop is located. We added the latitude and longitude of each outcrop to the figure caption.

Fig. 5 – I suggest to provide more distinct differences in colors (darker vs. lighter). Iʼm not sure they will be distinguishable in final version.

We added made the in-place sample colors darker to contract better with the lighter.

Fig. 5 and 6 – If 1 is the oldest and 3 is the youngest, maybe its better to change the axes. Put histograms in stratigraphic order on axis y and relative abundance on axis x. If appropriate change also Fig. 8.

Because there are multiple outcrops in both figures, and the goal of the figure is to compare the two collection methods, rather than changes through time, we have kept the figures the same.

*Additional comments from annotated manuscript file:*

explicitly mentioning lithology/facies (as you also discuss its potential impact later in the text) would also be good in this context - outcrop availability does not entirely cover it.

“lithology” was added to the text.

Sometimes - the rock types also makes it difficult (in case of limestones). The following authors investigated polish section:

“lithology” was added to the text here as well.

I agree, but some authors also study them in thin or polished sections for some purposes for exactly this reason:

Klug, C., Samankassou, E., Pohle, A., De Baets, K., Franchi, F., & Korn, D. (2018). Oases of biodiversity: Early Devonian palaeoecology at Hamar Laghdad, Morocco. Neues Jahrbuch für Geologie und Paläontologie-Abhandlungen, 290(1-2), 9-48.

It would be good to have some references discussing it or at least making a statement about this.

This paper was cited after mentioning the reasons why some would use this method.

It might be helpful to have a least some pictures of some of the specimens you studied to get an idea about their preservation/determination. One could for example integrate some images of the dominant brachiopod taxa in figure 6.

We have added specimen images to Figure 6, and additional images as supplemental material.

It would also be good to have some reference for these which are not your own. This reference illustrates it quite nicely - although for difference purposes.

As mentioned below the Lobza paper is not peer-reviewed and contains questionable methods, data, and conclusions. We prefer not to cite it.

Not really clear, please rephrase. Around all identifications based on the morphology - maybe be more specific which traits were necessary to assign to and count fossils in this group.

We added, “e.g., stick or fenestrate bryozoans.”

Be more specific - you mean half a whorl or presence of a particular traits. Please rephrase.

We added “For most gastropods, the shell spire (minus the protoconch) was chosen as the countable landmark, as long as enough spire whorls were present to identify the taxon (thus body whorls without the spire were not counted). For mollusks in which the early whorls were enveloped within later whorls (e.g., bellerophontoid gastropods and coiled cephalopods), the central portion of the shell was counted.”

It would be good to mention how this might impact on your data/results

We did not add any text here. This would not impact anything about the results. This is just a means of ensuring a taxon that is found in the sample is included in the analyses because it could be indicative of a specific ecosystem variable that would be lost if it were ignored.

it would be more appropriate to add example like bryozoans behind colonial and crinoids behind articulated taxa. Why not just add all investigated groups in one of these categories.

We changed the text to, “Colonial (e.g., bryozoans and corals) and easily disarticulated taxa (e.g., crinoids) were included”

what do you mean - it is a bit confusing as you later mostly discuss only the one related to on collector active in all localities/types of collection.

We conducted all the different analyses to be thorough. Because all the analyses show the same result, we focused on these certain ones to examine in depth. We added the following text in the discussion, “Because results for all six dataset comparisons demonstrate the same patterns and reveal similar statistical results from the PERMANOVA, we will discuss the possible reasons for our results and make interpretations based on the mollusk and brachiopod (all collectors combined) dataset, to avoid repetition throughout the discussion. We chose the mollusk and brachiopod dataset in particular because it had the highest PERMANOVA p-value (p = 0.003) of the six dataset comparisons and thus is the most conservative for testing the null hypothesis (that there were no differences between the multivariate paleocommunity results between collecting in-place and surface samples).”

I feel it would make the figure even nice if you would add small cut-out of the representative specimens of the brachiopod taxa.

We have added specimen images to Figure 6, and additional images as supplemental material.

Using a Permanova might also be good here for consistency...

Permanova tests for difference between two groups. The null hypothesis here is that there is a difference, so could create type 1 error if Permanova is used here. We could have added in a mantel or PROTEST (multivariate test of association), but we did not think the extra text and explanation would be necessary to get our point across that sample size did not drive the differences between the results from the two collection methods.

more accurate than what? please add

We added “than using surface-collected samples” to the first paragraph of the discussion.

It would even be better to actually test how significant/important differences are - behind visualization through NMDS.

Please see the response above as to why we did not conduct a Permanova on the subsampled datasets.

Not sure how this proves your point - how is the presence of sponges additional evidence for weathering - please rephrase and explain in greater detail.

The text was changed to “The presence of sponges, abundant in the Jacksboro Limestone but not documented in the Finis Shale, in previous studies [37,38] or in any of the in-place samples in the present study, is additional evidence of stratigraphic mixing due to weathering.”

I understand this is out of the scope of this paper, but mentioning that one can test the inlfuence of size in the future would be mentioned.

We added, “We plan to conduct future research projects to determine how the small size communities (< 2 mm) compare to the larger size communities (> 2 mm) and to the surface collected sample communities.”

***Review #2:***

Choice of references: It seems that the authors have chosen only US-based literature while there is much information on this and related topics in European papers. For example Cassian Formation studies (Nützel & Kaim 2014, Hausmann et al. 2015) the differences in bulk sediment vs. surface sampling strategy are discussed with different conclusions than yours.

First, our literature review was conducted based on three top international paleontology journals. Second, we included as many of reviewer 2’s and 3’s references as we thought were relevant to the current research. However, many of the suggested references (in particular Nützel & Kaim 2014, Hausmann et al. 2015) have different goals than the current study. They are trying to obtain information from the fossils and use different methods. They are both systematic studies that do not directly compare any methods. They are simply describing the taxa or groups of taxa in a qualitative way.

We could not find anything specific in either of these references that compared the results of the different methods. Because this was the goal of our study, we did not discuss these suggested references in our introduction or discussion sections.

Strategy of sampling: One major flaw I see is 2 mm cut-off in bulk sediment samples. It

potentially removes several small-sized taxa. Also changes considerably rank abundance. I personally use 0.375 mm mesh size to keep in the sample nearly all taxonomically identifiable mollusks. It is also worth mentioning that bulk sediment samples are advisable for silicified and phosphatized fossils (see e.g., Halamski et al. 2015 and Dzik 1994 respectively). Bulk sampling is also a natural choice for fine grained siliciclastics (black clays) where the majority of fossils is small-sized and well preserved while the rock itself is easy to disintegrate (e.g. Kaim 2001, 2011, 2012, Kaim & Sztajner 2012 among others)

Please see the response to reviewer 1’s first comment above as to why we use the size cutoff of 2mm.

Contamination: Another source of major bias is possible contamination from overlying beds. Couldnʼt it be overcome? You mentioned this possibility yourself but, really, comparison of contaminated (surface) vs. pristine (bulk) samples has a little sense in this respect.

This is the exact point of this research project. We are demonstrating that the surface cannot be used to exemplify the communities of the Finis Shale because the surface samples could be contaminated. The comparison is valid because surface-collection is an actual method used at these types of localities. The overlying Jacksboro Limestone is present as only a very thin cap at the outcrop of the Finis Shale, but our study empirically demonstrates that fossils from it may be found on the surface of the outcrop.

We did not add any text regarding this issue because we have already discussed this point in detail in the discussion.

Surface covered: You mentioned that surface collection comes from approx. 25-100 m2 area while bulk samples are collected from 0.1 m x 0.1 m = 0.01 m2 area that is an order of magnitude smaller. No wonder that you receive unequal sample sizes. Also by means of diversity (not all microenviroments occurring in 100 m2 will be represented by 0.01 m2 subsample. You should collect the bulk sample all over this 100 m2 area in order to get good representation.

It would not be possible to get a representative bulk, in-place sample from that large of an area. We would have to take too much material, which would take much more time to collect and process and money to transport. But we did use two different portions of the same outcrop that were even further from one another than 10 square meters, and the results of those in-place were different from any surface anywhere. So this would not influence the results.

To clarify this point, we added the following text to the Richness portion of the discussion, “Because of the greater surface area covered to gather the surface samples, it could be that the difference in richness, and possibly the differences in taxonomic composition and abundance, could be a result of the greater spatial area covered. We were able to take into account this possibility by collecting at two spots at the same locality, the east and west outcrops at the Jacksboro Spillway, which are about 200 meters apart. The assemblages from the in-place samples from two outcrops were much more similar to one another than to the surface samples of either outcrop, suggesting that the in-place assemblages do not vary considerably spatially. Even the Causeway outcrop samples, 200 meters away from the Spillway outcrops, grouped according to collection method.”

Subsampling: Why no rarefaction performed. It would nicely address the issue of diversity change after subsampling. Also why no rank abundance given?

The multivariate analyses and comparisons take into account all of the information that would go into a rank abundance distributions. Rarefaction was used to examine the fact that we needed to do the subsampling originally. But doing the actual splits and then analyzing and comparing the results is more thorough that the simulation that is rarefaction. Furthermore, the goal of our study is to examine practical collection methods, rather than characterize the paleocommunities themselves.

With all that said, if the editor sees fit, we are happy to add additional analyses and data in regards to the subsampling. But for the sake of parsimony, we think it is best to keep the subsampling portion the way it is because it drives home the point that the sample size is not driving the observed differences in collection methods.

Finally I would recommend using bulk sediment vs. surface sampling instead in-place vs. surface. The latter sounds cumbersome, isnʼt surface sampling also in-place?

No, the surface samples are not in-place. They are weathered out from somewhere the Finis Shale or possibly the overlying Jacksboro Limestone, as evidenced by our results. So, we kept our original nomenclature.

References:

We have added any suggested references that were relevant to some aspect of the paper. Most were descriptive paleontology or paleoecology, without similar research goals or analytical methods. So, they were not relevant to any discussion points, but did serve as additional background material for the introduction.

The reviewer seems to suggest that because they collected material from both in-place and from the surface that these studies could provide insight into the findings of this study. Because the collection methods are either not as systematic (or are unclear) or because different statistical methods were used, these suggested studies were not comparable enough to use in the discussion.

*Additional comments from annotated manuscript file:*

It seems that you took into account only US-based studies and discarded a wealth of European works highly relevant to this topic.

We have added all suggested references that were relevant to some aspect of this paper.

This strategy dominates in research on fine siliclastics where the vast majority of fossils is small-sized and the preservation is generally good (e.g. black clays etc.).

We have added the suggested reference that includes this method

This might be a major bias as for the diversity. There are several taxa, especially gastropods, which would go through the mesh. Not mentioning forams and ostracods. I personally use mesh size 0.3-0.4 mm.

Please see the response to reviewer 1’s first comment above as to why we use the size cutoff of 2mm.

why no rank abundance chart?

We used statistical methods typical in community paleoecology research. The multivariate analyses we conducted take into account taxonomic rank abundance along with composition and total abundance. Rank abundance is not in itself a statistical analysis. Thus, using multivariate visualizations and tests of differences are the best means of conveying the comparison of the two collection methods. Adding in RAC or rarefaction would just be adding in more figures and analyses that are already conveyed in the ordinations and Permanovas.

I think this term is introduces unnecessary complication, "bulk-sediment sample" is just enough.

We disagree, because you could bulk-sediment sample from the surface as well.

Why no rarefaction performed, it would address this problem nicely. It would show you what to expect after subsampling.

Rarefaction would simulate the subsampling procedure. The subsampling procedure using splits was completed in place of rarefaction in order to determine whether sample size was influencing the results.

Such contamination is a serious bias rendering the samples incomparable

This is the exact point of the research project. We are demonstrating that the surface can not be used to exemplify the communities of the Finis Shale because of this exact point.

This is really biasing your result. You should collect bulk samples all over this 10 square meters to keep the samples comparable. Otherwise the surface (and microenvironments) covered is an order of magnitude smaller in bulk sampling.

See response to this same comment above.

***Review #3:***

These mainly concern some additional references concerning influence of lithology on preservation/collection or a references summarizing diversity in the Finis Shale other than your own as well as some lithologies/localities (e.g., Liberation Lagerstatten) might be more prone to use the herein suggested methods.

Lobza, V., Schieber, J. & Merlynd, N. 1994. The Influence of Sea Level Changes and Possible Pycnocline Shifts on Benthic Communities in the Finis Shale (Virgilian) Near Jacksboro, North- Central Texas. Canadian Society of Petroleum Geologists Memoir, 17, 927-947.: This is non-peer reviewed and has some statistical and methodological insconsistencies. We have never references it in any of our publications on the Finis Shale, and I would prefer to keep it out of this one.

We have included the other two suggested publications.

You share all data but it is hard to get an idea about preservation and determination of fossils within seeing at least some pictures of the main taxa - at least in the supplementary material. Some of these could also be integrated in the figures (e.g., pictures of the main brachiopod species integrated in figure 6).

We have added specimen images to Figure 6, and additional images as supplemental material.

In some cases, the NMDS labels (without points) are also hard to read (e.g., overlapping in Figs. 3 and 4). Some additional multivariate tests (e.g., Permanova as you did for the others) supporting the NMDS results for the subsampling might also be appropriate for the sake of consistency or would these not be appropriate in this case?

We created new NMDS ordinations for both figures 3 and 4 with much more clearly visible points in place of the text in the ordination.

Permanova tests for difference between two groups. The null hypothesis here is that there is a difference, so could create type 1 error if Permanova is used here. We could have added in a mantel or PROTEST (multivariate test of association), but we did not think the extra text and explanation would be necessary to get our point across that sample size did not drive the differences between the results from the two collection methods.
