# Supplementary material for: The influence of collection method on paleoecological datasets: In-place versus surface-collected fossil samples in the Pennsylvanian Finis Shale, Texas, USA
Source: PLoS One. 2020 Feb 11;15(2):e0228944. doi: 10.1371/journal.pone.0228944 (PMC7012410; doi:10.1371/journal.pone.0228944)
Supplement: Attachment — Submitted filename: Forcino and Stafford Response to Reviewers Comment.docx [file pone.0228944.s007.docx]

I would like to ask you to consider a small development of conclusions - in the present version they are very general, while your manuscript brings large amount of quantitative data. Therefore I would suggest you to include in the conclusions and abstract some quantitative comparisons between bulk- and surface sampling.

We have added text to the abstract and conclusions that includes our statistical results.
